# Characteristics of Vaginal Microbiota of Women of Reproductive Age with Infections

**DOI:** 10.3390/microorganisms12051030

**Published:** 2024-05-20

**Authors:** Wanting Dong, Siyi Wang, Xi Wang, Guojin Xu, Qiuying Liu, Zheng Li, Na Lv, Yuanlong Pan, Qian Xiong, Donglai Liu, Baoli Zhu

**Affiliations:** 1 CAS Key Laboratory of Pathogenic Microbiology and Immunology, Institute of Microbiology, Chinese Academy of Sciences, Beijing 100101, China; dongwt@im.ac.cn (W.D.);; 2 University of Chinese Academy of Sciences, Beijing 100049, China; 3 Division II of In Vitro Diagnostics for Infectious Diseases, Institute for In Vitro Diagnostics Control, National Institutes for Food and Drug Control, Beijing 100050, China; 4 NMPA Key Laboratory for Quality Research and Evaluation of Medical Devices, Beijing 100050, China; 5 NMPA Key Laboratory for Quality Research and Evaluation of In Vitro Diagnostics, Beijing 100050, China; 6 Department of Pathogenic Biology, School of Basic Medical Sciences, Southwest Medical University, Luzhou 646000, China; 7 Jinan Microecological Biomedicine Shandong Laboratory, Jinan 250117, China; 8 Beijing Key Laboratory of Antimicrobial Resistance and Pathogen Genomics, Beijing 100101, China

**Keywords:** vaginal microbiota, 16S rRNA gene sequencing, vaginal infections, vaginal dysbiosis

## Abstract

The vaginal microbiota can be classified into five major community state types (CSTs) based on the bacterial content. However, the link between different CST subtypes and vaginal infection remains unclear. Here, we analyzed 2017 vaginal microbiota samples from women of a reproductive age with vaginal infections that were published in the last decade. We found that *L. iners* was the most dominant in 34.8% of the vaginal samples, followed by *L. crispatus* (21.2%). CST I was common in healthy individuals, whereas CST III and IV were associated with dysbiosis and infection. CST III-B, IV-A, IV-B, and IV-C0 were prevalent in patients with bacterial vaginosis (BV). Based on the relative abundance of bacteria at the (sub)genus level, a random forest classifier was developed to predict vaginal infections with an area under the curve of 0.83. We further identified four modules of co-occurring bacterial taxa: *L. crispatus*, *Gardnerella*, *Prevotella*, and *Bacteroides*. The functional prediction revealed that nucleotide biosynthesis pathways were upregulated in patients with human papilloma virus, and carbohydrate degradation pathways were downregulated in patients with BV. Overall, our study identified the bacterial signatures of healthy and infected vaginal microbiota, providing unique insights into the clinical diagnosis and health status prediction of women of a reproductive age.

## 1. Introduction

The vaginal microbiota of healthy women is dominated by a single *Lactobacillus* species [1]. In general, *Lactobacillus* spp. inhibits the proliferation of exotic bacterial species, such as opportunistic pathogens, in the vagina by producing lactic acid, hydrogen peroxide, and bacteriocins [2]. In dysbiosis, other facultative or strictly anaerobic microorganisms such as *Gardnerella* and *Prevotella* spp. can also inhabit the vagina [3].

To better illustrate the structure of the vaginal microbiota, a clustering algorithm based on the composition and relative abundance of vaginal bacteria at the species level, called community state types (CSTs), was proposed [4], in which the vaginal microbiota was classified into five CSTs based on four *Lactobacillus* species. CST I was dominated by *L. crispatus*, CST II by *L. gasseri*, CST III by *L. iners*, and CST V by *L. jensenii.* CST IV was exceptional, because it was not dominated by any *Lactobacillus* species; instead, it consisted of different anaerobic species, such as *Gardnerella vaginalis* and *Prevotella spp.* CST IV was classified into three independent CSTs based on the type of dominant anaerobic species: CST IV-A, CST IV-C, and CST IV-D [5]. Since then, CSTs have been widely used to describe the vaginal microbiome, which may be associated with the health status of the female reproductive system. Recently, the CST scheme has been extended to 13 subtypes named VALENCIA, which is short for the VAginaL community state typE Nearest CentroId clAssifier [6], where CST I is classified into CST I-A and CST I-B, CST III is classified into CST III-A and CST III-B, and CST IV is classified into CST IV-A, IV-B, IV-C0, IV-C1, IV-C2, IV-C3, and IV-C4. CST IV-A is represented by a high to moderate abundance of *BVAB1* and *G. vaginalis*; CST IV-B is represented by a high to moderate abundance of *G. vaginalis* and *A. vaginae*; and CST IV-C1, C2, C3, and C4 are dominated by *Prevotella* spp., *Enterococcus* spp., *Bifidobacterium* spp., and *Straphylococcus* spp., respectively. This new classification can be applied to any cohort study to allow for comparative analysis between studies.

The composition of the vaginal microbiota is dynamic and influenced by ethnicity, sexual activity, age, and lifestyle [7]. More specifically, the predominant CSTs among different ethnic groups differ significantly, with African and Hispanic women having a lower abundance of *Lactobacillus* and a higher proportion of CST IV than Caucasian and Asian women [8]. Estrogen levels, throughout the life of women, starting from baby, child, puberty, and reproductive age (menstruation and pregnancy) to menopause, promote changes in the vaginal microbiota, as they are correlated with the abundance of *Lactobacillus* spp. and the vaginal pH [9,10,11,12,13]. CST Ⅰ, CST III, and CST IV are widely observed in women of a reproductive age, whereas CST V is uncommon [14,15]. Large cohort studies in Europe and North America have investigated the characteristics of vaginal microbiota composition [6,16]. The lack of systematic studies from Africa, Asia, and Oceania has resulted in a failure to provide a global baseline for a healthy vaginal microbiota.

In recent years, the association between a disturbed vaginal microbiota and gynecological and obstetric outcomes has been widely studied, especially bacterial vaginosis (BV) [17,18,19], sexually transmitted infections (STIs) [20,21,22], and preterm births [5,23]. CST IV consists of various strictly anaerobic bacteria, such as *Gardnerella vaginalis*, *BVAB1/2/3*, *Prevotella* spp., *Atopobium* spp., and *Megasphaera* spp., and is associated with different types of vaginal dysbiosis or infections, including aerobic vaginitis (AV), BV, STIs such as human papilloma virus (HPV), herpes simplex virus (HSV), and *Chlamydia trachomatis* (CT) infections, and vulvovaginal candidiasis (VVC) [24]. However, CST IV-associated infections do not always present with symptoms [25], raising questions about whether CST IV or any subtype of CST IV increases the risk of adverse health outcomes.

The accumulated sequencing data of the vaginal microbiota enabled us to explore the features of the vaginal microbiota under different health conditions. Here, we present a combined analysis of 16S rRNA amplicon sequencing data of 2017 vaginal microbiomes from 14 studies to investigate dysbiosis or infection-associated microbial signatures, thus contributing to the overall understanding of the healthy vaginal microbiota and future diagnosis of vaginal infection with microbial signatures.

## 2. Materials and Methods

### 2.1. Data Sources and Study Population

The 16S rRNA gene sequencing data of vaginal microbiota from women with different types of vaginal infection and healthy women were downloaded by searching published research using the keywords “16S rRNA gene sequencing and vagina”. The inclusion criteria for the studies were as follows: (1) studies containing publicly available 16S rRNA gene sequencing data using Illumina sequencing platforms, (2) studies using vaginal swabs as sample sources, and (3) studies containing publicly available metadata, especially group information for each sample. A total of 14 studies with accessible raw data and metadata of vaginal samples were selected for this study [22,26,27,28,29,30,31,32,33,34,35,36,37,38]. For longitudinal studies in which women were treated with probiotics or medicines after diagnosis, only the baseline samples were selected. In total, 2017 samples from healthy individuals and six types of infections from different areas were used in our analyses. The details of the included studies and their samples are presented in Table 1.

### 2.2. 16S rRNA Data Processing

Because the targeted regions of the 16S rRNA gene sequencing data downloaded from public databases were the V3–V4 or V4 regions, the V4 region of these sequences was extracted for integrated analysis. Briefly, the pair-end reads were merged into long sequences using QIIME2 (version 2020.8.0) [39]. The V4 regions were extracted using Cutadapt (version 2.3) by marking the 16S rRNA sequences of the 515F and 806R primers with single-end reads or merged pair-end reads [40]. Subsequently, the deblur plugin in QIIME2 was used to denoise the sequencing reads and generate amplicon sequence variants (ASVs). Next, the ASVs were taxonomically classified using the downloaded naïve Bayes classifier, trained using the 16S rRNA V4 sequences in the SILVA database (silva-138-99-515-806-nb-classifier-download) for each dataset. Samples with less than 1000 mapped reads were filtered for analysis.

### 2.3. Lactobacillus Subgenus Reclassification

ASVs generated by 16S rRNA gene sequencing can only be accurately classified to the genus level, whereas the analysis of vaginal *Lactobacillus* requires species-level classification. Therefore, we reclassified the ASVs of *Lactobacillus* into subgenera according to the method proposed by France et al. [16]. Briefly, species of the *Lactobacillus* genus were reclassified into nine subgenera based on the maximum-likelihood phylogenetic tree, constructed using amino acid sequences of 100 single-copy core genes of representative genomes of all *Lactobacillus* species. This method can differentiate the four major vaginal *Lactobacillus* species into different *Lactobacillus* subgenera.

### 2.4. VALENCIA Clustering Analysis

The VALENCIA is a clustering scheme based on the nearest centroid classification algorithm, which was used for reproducible and rigorous classification of vaginal microbiota [6]. The vaginal microbiota was classified into 5 CSTs: CST I (*L. crispatus*- dominated), II (*L. gasseri*-dominated), III (*L. iners*-dominated), IV (Non-*Lactobacillus*-dominated), and V (*L. jensenii*-dominated). The CSTs were further classified into 13 subtypes based on the percentage and type of dominant species. Specifically, CST I was classified into CST I-A and I-B, CST III was classified into CST III-A and III-B, and CST IV was classified as CST IV-A, IV-B, IV-C0, IV-C1, IV-C2, IV-C3, and IV-C4. To map our data to the CSTs defined by VALENCIA, we manually modified the subgenus of *Lactobacillus* into the VALENCIA taxon names.

### 2.5. t-SNE Analysis of the Vaginal Microbiota

To investigate the structure of the vaginal microbiota within our samples, t-SNE was performed on the relative abundances of the (sub)genus in each sample using the Bray–Curtis distance metric to calculate distances using the TSNE function in the sklearn.manifold module. Samples were labeled with CSTs, CST subtypes, the most dominant taxa, the second most dominant taxa, and the abundances of the most and second most dominant taxa.

### 2.6. Network Analysis

To explore the correlations between vaginal taxa, we performed a network correlation analysis using SparCC with 1000 bootstrap replicates to estimate the *p*-values [41]. Only species that were present in at least 10% of all samples were retained for network analysis. The network was visualized using the igraph package in R.

### 2.7. Functional Prediction of the Microbiota

The relative abundances of the (sub)genera were calculated based on the ASV tables. The metagenomic functional composition of the vaginal microbiota was predicted based on the relative abundance of the (sub)genus using PICRUSt2 [42]. The relative abundance of metabolic pathways was annotated using the MetaCyc database. The relative abundances of pathways between the healthy and disease groups were compared using the ggpicrust2 package in R. Statistical analysis was performed using the embedded DESeq2 method.

### 2.8. Random Forest Model

To assess the performance of vaginal microbiota in predicting vaginal infection, random forest models were trained based on the relative abundance of vaginal microbiota in each sample using the RandomForest Classifier function in the sklearn.ensemble module. First, all the samples were randomly split into training (70%) and test datasets (30%). To obtain the best (sub)genus for classification and the best combination of hyperparameters in each model, feature and hyperparameter selections were performed and embedded into a random search pipeline with 10-fold cross-validation. Finally, the performance of the model was evaluated using the area under the curve (AUC), accuracy, precision, and sensitivity, which were calculated using roc_auc_score, accuracy_score, precision_score, and recall_score functions in the sklearn.metrics module, respectively.

### 2.9. Statistical Analysis

Intergroup comparisons were performed using Wilcoxon rank sum tests in R. BH adjustment was used to control the FDR in multiple hypothesis tests. All statistical tests were two-sided. *p*-values < 0.05 were regarded as significant after adjusting for multiple testing.

## 3. Results

### 3.1. Lactobacillus Species Were Dominant in the Vagina

**Figure 1:**
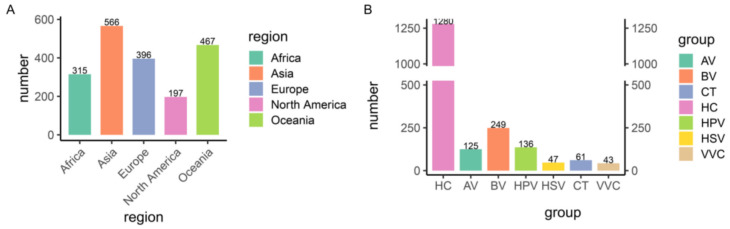
Overview of the 1941 samples. (**A**) Geographic distribution of the samples. (**B**) Number of samples in each disease group. HC, healthy control; AV, aerobic vaginitis; BV, bacterial vaginosis; VVC, vulvovaginal candidiasis; CT, Chlamydia trachomatis; HPV, human papilloma virus; HSV, herpes simplex virus.

We collected 2017 sequencing data of the 16S rRNA gene of vaginal swabs from 14 studies to characterize the vaginal microbiota of women of a reproductive age (Table 1), including samples from healthy controls and aerobic vaginitis (AV), bacterial vaginosis (BV), vulvovaginal candidiasis (VVC)-, Chlamydia trachomatis (CT)-, human papilloma virus (HPV)-, and herpes simplex virus (HSV)-infected individuals from different continents. After sequencing quality assessment, a set of 1941 samples was selected for further analysis, including 315 samples from Africa, 566 from Asia, 396 from Europe, 197 from North America, and 467 from Oceania (Figure 1A,B).

**Figure 2:**
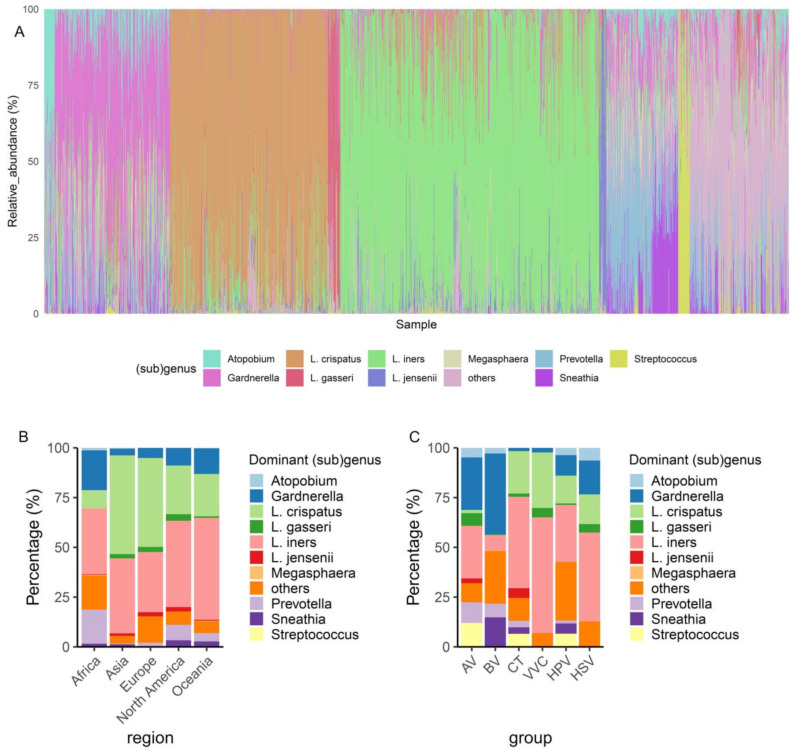
Overview of the dominant taxa in the vaginal microbiota. (**A**) The relative abundance of the vaginal microbiota in each sample. (**B**) The distribution of the most dominant taxa in each region of healthy individuals. (**C**) The distribution of the most dominant taxa in each disease group. AV, aerobic vaginitis; BV, bacterial vaginosis; VVC, vulvovaginal candidiasis; CT, Chlamydia trachomatis; HPV, human papilloma virus; HSV, herpes simplex virus.

For comparative analysis, we used sequences of the V4 region of the 16S rRNA gene to profile the vaginal microbiota. Subsequently, the V4 region sequences of the *Lactobacillus* genus were reclassified to the subgenus level, resulting in nine subgenus groups that included the *L. crispatus*, *L. iners*, *L. gasseri*, and *L. jensenii* groups, and five other uncommon groups, the *L. delbrueckii*, *L. pasteurii*, *L. apis*, *L. sp002417825*, and *L. sp002418055* groups [16]. The results of the comparative analysis showed that the *Lactobacillus* genus was dominant in the vaginal microbiota by 58.6%, with *L. iners* group being the most prevalent (34.8%), followed by the *L. crispatus* (21.2%), *L. gasseri* (1.6%), and *L. jensenii* groups (1.0%) (Figure 2A); the genus *Gardnerella* occupied 15.5%, followed by *Prevotella* (6.1%), *Sneathia* (3.4%), *Streptococcus* (1.5%), *Atopobium* (1.4%), and *Megasphaera* (0.15%), and the remaining genera occupied 13.3% (Figure 2A). 

Among healthy individuals, *L. crispatus* was dominant in Asia and Europe, whereas *L. iners* was dominant in Africa, North America, and Oceania (Figure 2B). The percentages of *Gardnerella*- and *Prevotella*-dominated vaginal microbiota were higher in Africa than in other regions (Figure 2B). Among the vaginal microbiota with infections, the dominance of *L. iners* was widely identified in the CT, VVC, HPV, and HSV patient groups, indicating that *L. iners* was susceptible to vaginal infections. A *L. crispatus*-dominated microbiota was the second most common after *L. iners* in the CT, VVC, HPV, and HSV patient groups, whereas a *Gardnerella*-dominated microbiota was less common in these patient groups (Figure 2C). A *Gardnerella*-dominated microbiota was the most prevalent in patients with AV and BV, followed by *L. iners* and *Prevotella* (Figure 2C). An *L. crispatus*-dominated microbiota was absent in patients with BV. In summary, the dominant bacterial groups in healthy individuals from different continents differed, with *L. cripatus* and *L. iners* being the most common and dominant taxa. An *L. iners*-dominated microbiota was also common in patients with CT, VVC, HPV, and HSV infections, and *Gardnerella* was enriched in patients with BV. 

### 3.2. CST III-A Was Enriched in Patients with Vaginal Infections

**Figure 3:**
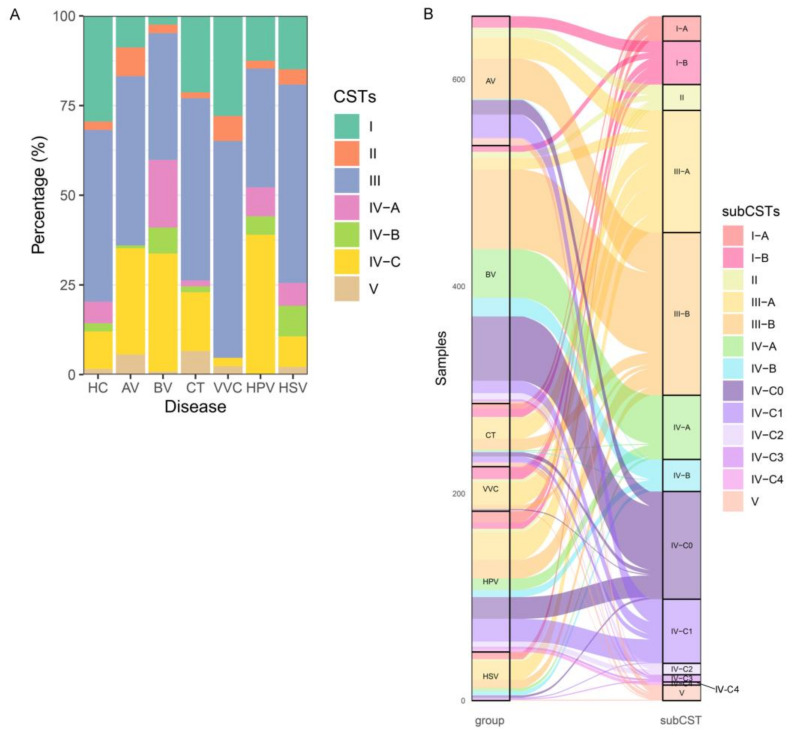
Overview of the CSTs in the vaginal microbiota. (**A**) The CSTs of the vaginal microbiota in each sample. (**B**) The distribution of CST subtypes in each disease group. HC, healthy control; AV, aerobic vaginitis; BV, bacterial vaginosis; VVC, vulvovaginal candidiasis; CT, Chlamydia trachomatis; HPV, human papilloma virus; HSV, herpes simplex virus.

Next, we compared the subtypes of vaginal microbiota between different groups based on VALENCIA, which is a newly developed CST classifier. The 13 subtypes of CSTs defined by VALENCIA were identified in 1941 samples. The prevalence of CST I was much higher in healthy women than in the patient groups, except for VVC, whereas the CST III and IV were more prevalent in the patient groups (Figure 3A). The enrichment of CST III varied in different diseases: CST III-B was enriched in patients with AV and BV, whereas CST III-A was enriched in patients with CT, VVC, HPV, and HSV. In contrast, CST IV was more enriched in patients with BV and HPV. Notably, CST IV-A, IV-B, and IV-C0 were more prevalent in patients with BV (Figure 3B). Thus, we inferred that *G. vaginalis*-dominated CSTs, such as CST IV-A and IV-B, were representative of patients with BV, and that women with CST III-A were more likely to develop vaginal infections.

### 3.3. CST III-B Components Were Complex and Associated with Patients with BV

**Figure 4:**
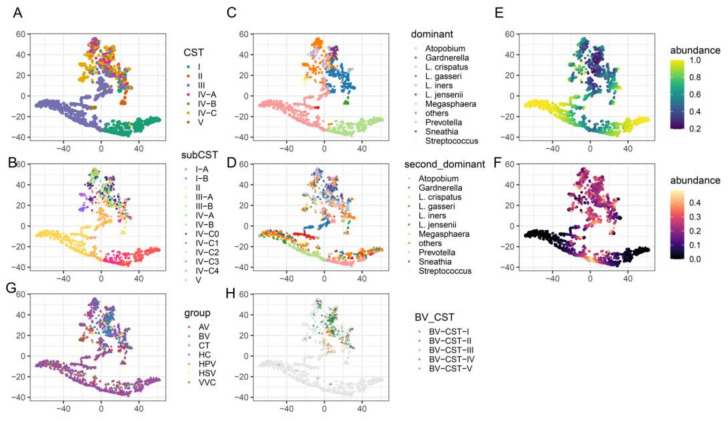
t-SNE plot of microbiome samples. Samples are colored according to the CSTs (**A**), 13 subtypes of CSTs (**B**), dominant taxa (**C**), second most dominant taxa (**D**), abundance of the dominant taxa (**E**), abundance of the second most dominant taxa (**F**), disease group (**G**), and CSTs of the BV samples (**H**). AV, aerobic vaginitis; BV, bacterial vaginosis; VVC, vulvovaginal candidiasis; CT, Chlamydia trachomatis; HPV, human papilloma virus; HSV, herpes simplex virus.

The enrichment of CST III in both healthy individuals and individuals with a disease prompted us to compare the vaginal microbiota structures of these samples. The t-SNE analysis showed that *Lactobacillus*-dominated CST samples formed one cluster and were separated from the cluster formed by non-*Lactobacillus*-dominated CST samples. However, a group of CST III-B samples was found between the two clusters (Figure 4A,B). CSV III-B was originally defined as the vaginal microbiota in which *L. iners* was less abundant but still dominant; however, three subgroups of CST III-B were observed under t-SNE analysis that we further assigned as CST III-B0, III-B1, and III-B2. In CST III-B0, *L. iners* and *Gardnerella* were co-dominant with comparable relative abundances (Figure 4C–F). The CST III-B1 samples, which consisted of *Gardnerella*, *Sneathia*, *Prevotella,* and other non-*Lactobacillus* genera, merged into the cluster formed by the non-*Lactobacillus*-dominant CST samples, indicating that these samples were not properly clustered or defined by VALENCIA (Figure 4B–F). Most CST III-B1 samples belonged to the BV group, explaining why CST III-B was enriched in patients with BV (Figure 4G,H). The intermediate group between CST I-B and III-A, which we assigned as CST III-B2, was co-dominated by *L. iners* and *L. crispatus,* with relative abundances ranging from 40% to 60% (Figure 4B–F). Therefore, patients with BV tend to process *Gardnerella*-related vaginal microbiota compared with healthy individuals and patients with other types of infections. 

### 3.4. The Potential Use of a Prediction Model Based on the Vaginal Microbiota in Clinical Diagnosis

To explore the potential of diagnosing vaginal infections based on the composition of the vaginal microbiota, a random forest model was employed to distinguish between vaginal dysbiosis and infections in healthy individuals. We constructed a random forest model with a relative abundance of vaginal microbiota and clinical metadata, which achieved good performance on the test dataset, as indicated by an AUC of 83% (Figure S1, Table S1). The accuracy and precision of the model were 79% and 76%, respectively, and the sensitivity of the model was 57%. The low sensitivity indicated that vaginal infections were difficult to diagnose when only the relative abundance of the vaginal microbiota was provided. However, the accuracy, precision, and sensitivity were improved when we used the model for BV prediction alone, with 92%, 83%, and 67%, respectively, indicating that the characteristics of the vaginal microbiota of patients with BV were more distinguishable than those of other vaginal infections (Figure S2, Table S2).

### 3.5. Functional Profiling of Metabolic Pathways in Vaginal Microbiota of Patient Groups

**Figure 5:**
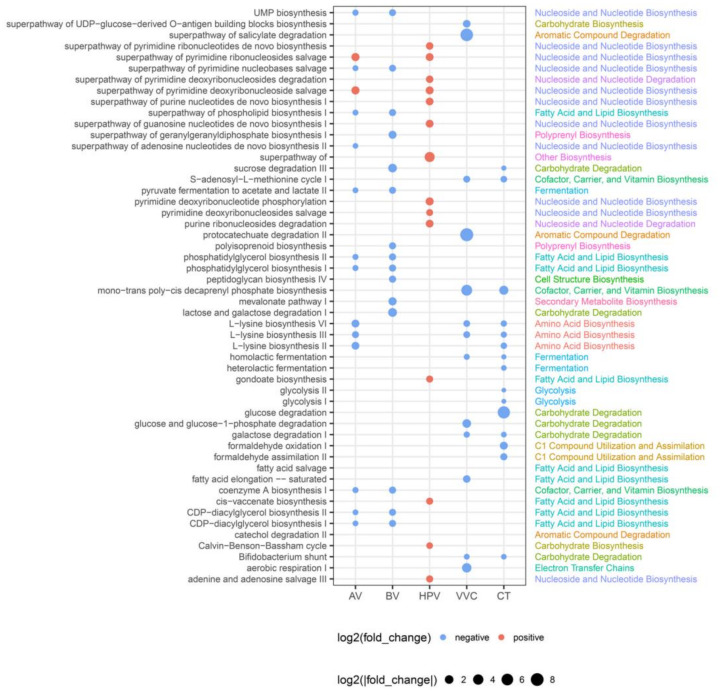
Differential functional pathways between the disease group and healthy controls. The size of the dot represents |log2(fold change)|. If the dots are red, the pathway is upregulated in the disease group. If the dots are blue, the pathway is downregulated in the disease group. Only pathways with *p* < 0.05 are shown. AV, aerobic vaginitis; BV, bacterial vaginosis; VVC, vulvovaginal candidiasis; CT, Chlamydia trachomatis; HPV, human papilloma virus.

We further investigated the metabolic profiles of the vaginal microbiota in each disease group by performing metabolic pathway annotations using the MetaCyc database. Of the 1941 samples, 1372 were successfully annotated with at least one pathway. All HSV samples failed to be annotated to any pathway and were not used for subsequent comparative analysis, which was performed between the healthy group and each patient group. A total of 1071 differential pathways were identified, of which 218 were identified in the AV group, 282 in the BV group, 193 in the HPV group, 243 in the VVC group, and 135 in the CT group (*p* < 0.05). Specifically, the nucleotide biosynthesis and degradation pathways (de novo purine nucleotide biosynthesis and pyrimidine nucleotide salvage) were significantly elevated in the HPV group (Figure 5). The VVC and CT groups shared several downregulated pathways, including amino acid biosynthesis (L-lysine biosynthesis), carbohydrate degradation (galactose degradation), and fermentation (fermentation to lactate) (Figure 5). Moreover, the AV and BV groups showed similar patterns of metabolic profiles, where fatty acid and lipid biosynthesis pathways, such as phosphatidylglycerol biosynthesis and CDP-diacylglycerol biosynthesis, carrier biosynthesis pathways, such as coenzyme A biosynthesis, and pyruvate fermentation pathway were all downregulated. The lactose and sucrose degradation pathways were exclusively downregulated in the BV group (Figure 5). Overall, the differential pathways in HPV-infected individuals were characterized by enhanced nucleotide biosynthesis pathways, whereas carbohydrate metabolic pathways were downregulated in patients with VVC and CT and fatty acid, and lipid biosynthesis pathways were downregulated in patients with AV and BV.

### 3.6. Colonization Resistance Can Be Explained by Bacterial Co-Occurrence Modules

**Figure 6:**
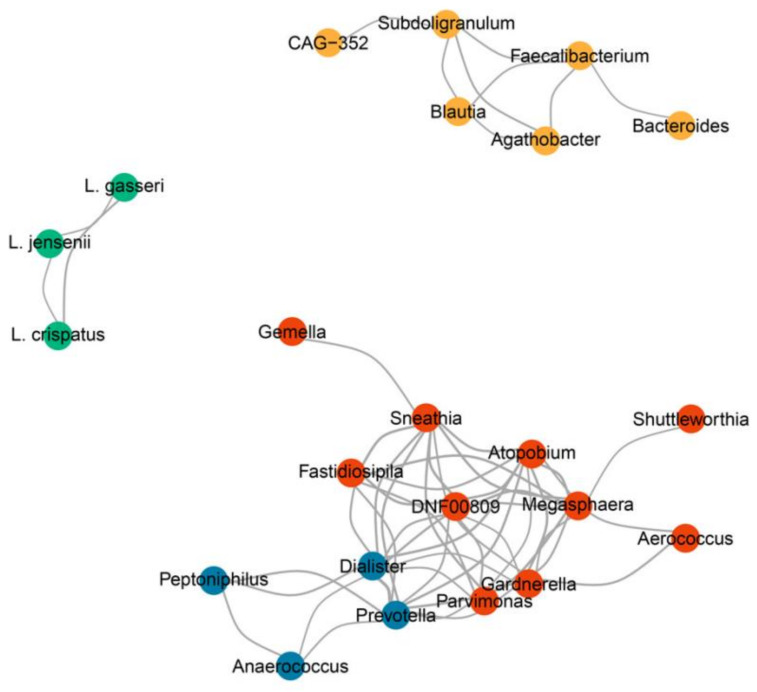
Co-occurrence network of the vaginal microbiota. Four modules of the correlated vaginal microbiota were identified: *L. crispatus* (green), *Gardnerella* (red), *Prevotella* (blue), and *Bacteroides* (yellow) modules. The line thickness represents the degree of correlation. Only correlations between subgenera with |r > 0.4| and *p* < 0.05 are shown.

Since we observed that *L. iners* co-occurred with different vaginal bacteria and was enriched in both healthy and disease groups, we investigated how different bacterial taxa in the vagina were correlated. Four bacterial co-occurrence modules of the correlated bacterial taxa were identified: *L. crispatus*, *Gardnerella*, *Prevotella,* and *Bacteroides* (Figure 6). Within the *L. crispatus* module, *L. crispatus* was strongly correlated with *L. jensenii* and *L. gasseri* (r = 0.4 and 0.5, *p* < 0.05). The bacterial taxa of the *L. crispatus* module were negatively correlated with the bacterial taxa in the *Gardnerella* and *Prevotella* modules (r between −0.25 and −0.04 and between −0.31 and −0.07, respectively) and positively correlated with the bacterial taxa of the *Bacteroides* module (r = 0.04–0.2). In addition, the bacterial taxa in the *Prevotella* module were positively correlated with the taxa in the *Gardnerella* module, except that *Anaerococcus* in the *Prevotella* module showed a negative correlation with *Shuttleworthia* in the *Gardnerella* module (r  =  −0.12–0.64). Interestingly, the *L. iners* group did not correlate with any bacterial taxa, indicating that the *L. iners* group coexisted with any bacterial taxa, which explains the three subgroups observed in CST III-B. In summary, the *L. crispatus* module showed evidence of colonization resistance to *Gardnerella* and *Prevotella*. However, *L. iners* did not show colonization resistance to any bacterial taxa in the vagina.

## 4. Discussion

The vaginal microbiota plays an important role in the health of women of a reproductive age [43,44]; however, the association between the vaginal microbiota and different types of vaginal infections is not clear. In this study, we systematically analyzed the characteristics of the vaginal microbiota of 1280 healthy and 661 infected women of a reproductive age. Unlike previous studies, we aimed to decipher the bacterial co-occurrence modules in the vaginal microbiota associated with infections to define the colonization resistance. The results were based on an assessment of previous studies of patients with six types of dysbiosis or infections in comparison with healthy individuals. 

The *L. iners*-dominated vaginal microbiota was prevalent (34.8%) in both the healthy individuals and the disease groups and was observed in women of a reproductive age in Africa, North America, and Oceania, with the highest prevalence. The CST analysis further verified the enrichment of CST-III in the patient groups, where CST III-A was enriched in the VVC, CT, and HSV patient groups, and CST III-B was enriched in the BV patient group. Notably, in our study, CST III-B could be reclassified into three subgroups by t-SNE analysis, including CST III-B0, III-B1, and III-B2, owing to the differences in co-inhabitant bacterial taxa. CST III-B0 had *L. iners* cohabitating with *G. vaginalis* with comparable relative abundances; CST III-B1 was not dominated by *L. iners* but was composed of different types of non-*Lactobacillus* species; and CST III-B2 had *L. iners* cohabitating with *L. crispatus* with comparable relative abundances. CST III-B0 was previously described as a transition from *L. crispatus* to *L. iners* [12]. However, we inferred that CST III-B1 is a new subtype of CST IV that cannot be properly classified using VALENCIA. VALENCIA was constructed based on the vaginal microbiota of North American cohorts [6], while our data consisted of individuals from different continents and with different health statuses, thus covering more diverse vaginal microbiota or non-*Lactobacillus*-dominated vaginal microbiota. 

In terms of colonization resistance, we showed the ability of *L. iners* to cohabit with either *L. crispatus,* regarded as “good” vaginal bacteria [45,46], or *G. vaginalis,* regarded as “bad” vaginal bacteria [47,48]. *L. iners* did not show any positive or negative correlation with other vaginal genera in our co-occurrence network analysis or in a previous study [24]. We assumed that *L. iners* did not show colonization resistance to other vaginal bacterial taxa. In contrast, *L. crispatus* showed clear co-occurrence patterns with *L. jensenii* and *L. gasseri* and showed colonization resistance to *Gardnerella* and *Prevotella*, reinforcing the concept that *L. crispatus* contributes to a healthy and stable vaginal microbiota [45,49]. The features that the *L. iners* can co-inhabit with “good” *L. crispatus* or “bad” *G. vaginalis* is probably the reason why CST III-B was enriched in both healthy and infected individuals. We hypothesized that *L. iners* is a neutral bacterium, and its contribution to health depends on the cohabiting bacteria. The initial colonization of *Gardnerella spp.* in *L. iners*-dominated microbiota was more likely to develop into BV than in *L. crispatus*-dominated microbiota. Thus, the presence of *L. iners* is an alarming sign that the vaginal microbiota may have an unhealthy outcome. One possible explanation is that *L. iners* shares certain metabolic and physical interactions with *L. crispatus* and *G. vaginalis*. Another interpretation is that the strains of *L. iners* show divergent metabolic potential that can be shared with *L. crispatus* or *G. vaginalis*.

In previous studies, the association between *G. vaginalis* and BV has not been determined, because *G. vaginalis* has been detected in asymptomatic patients with BV, non-sex-experienced adolescent girls [11], and healthy women [25]. In our cohort, 41% of the vaginal microbiota in patients with BV was dominated by *Gardnerella*, followed by *Sneathia* (15%), *L. iners* (8%), *Prevotella* (7%), and *Atopobium* (3%). Moreover, CSTs dominated by *G. vaginalis* (CST-IV A and IV B) were more abundant in patients with BV than in those with other types of vaginal infection. The abundance and prevalence of *G. vaginalis* were lower in healthy individuals than in patients with BV, implying that the abundance, rather than the presence of *G. vaginalis,* is more important for BV diagnosis. Combining Nugent scores [50], Amsel criteria [51], and our diagnostic model may further improve the accuracy of clinical diagnosis of BV. Furthermore, our co-occurrence analysis revealed a positive correlation within members of the *Gardnerella* module and between the *Gardnerella* and *Prevotella* modules, which indicated that the colonization of non-*Lactobacillus* species would promote the colonization of other non-*Lactobacillus* species. 

Compared to BV, vaginal infections such as VVC, CT, and HSV did not show a specific pattern of vaginal dysbiosis but showed a higher percentage of *Lactobacillus iners*-dominated microbiota (CST III-A), indicating that the recovery of vaginal microbiota from non-*Lactobacillus*-dominated or *L. iners*-dominated to *L. crispatus*-dominated is essentially important to decrease recurrence and for the successful treatment of vaginal infections. The diagnostic model for predicting vaginal dysbiosis and infections did not show as good a performance as that predicting BV. We inferred that the (sub)genus-level profiling of vaginal microbiota using 16S rRNA gene sequencing is not enough for differentiating healthy vaginal microbiota and infected microbiota. In future studies, strain-level or species-level analysis of vaginal microbiota should be performed by using metagenomic sequencing to decipher the presence of different *Lactobacillus* strains in vagina microbiota and their contribution to women’s reproductive health.

## 5. Conclusions

In conclusion, the vaginal microbiota of healthy individuals and patients were characterized by different bacterial co-occurrence modules. The vaginal microbiota in CST-I, dominated by *L. crispatus*, was more stable and enriched in healthy individuals, showing a higher colonization resistance to *Gardnerella* and *Prevotella*. In contrast, CST-III, which was dominated by *L. iners,* showed no co-occurrence modules with no colonization resistance and was susceptible to infections; for example, CST III-A was enriched in patients with HPV, HSV, CT, and VVC, whereas CST III-B was enriched in patients with BV. BV is a typical dysbiosis with higher *Gardnerella*, dominated by CST subtypes, such as CST IV-A, IV-B, IV-C0, CST III-B0, and CST III-B1.

## Figures and Tables

**Table 1:** Characteristics of the datasets in this study.

BioProject	No. Disease	No. Healthy	Group ^2^	Region ^1^	Reference
PRJNA511717	80	160	AV	V4	[26]
PRJEB29686	94	48	AV, BV	V4	[27]
PRJNA398590	20	-	BV	V3–V4	[28]
PRJNA592384	16	411	BV	V3–V4	[28]
PRJNA735440	27	-	BV	V3–V4	[28]
/	131	41	BV	V3–V4	[29]
PRJNA310998	49	1	BV, HSV	V3-V4	[22]
PRJNA523312	58	21	BV, CT, VVC	V3-V4	[30]
PRJEB63251	-	202	HC	V4	[31]
PRJNA391337	-	71	HC	V4	[32]
PRJNA730929	-	316	HC	V4	[33]
PRJNA518153	59	18	HPV	V4	[34]
PRJNA826816	32	-	HPV	V3–V4	[35]
PRJNA831622	45	-	HPV	V3–V4	[36]
PRJNA566293	42	35	CT	V3–V4	[37]
PRJEB33108	40	-	VVC	V4	[38]

^1^ Region of 16S rRNA gene. ^2^ HC, healthy control; AV, aerobic vaginitis; BV, bacterial vaginosis; VVC, vulvovaginal candidiasis; CT, Chlamydia trachomatis; HPV, human papilloma virus; HSV, herpes simplex virus.

## Data Availability

The public data used in this study are available in the NCBI SRA database via the NCBI BioProject ID PRJNA511717, PRJEB29686, PRJNA398590, PRJNA592384, PRJNA735440, PRJNA310998, PRJNA523312, PRJEB63251, PRJNA391337, PRJNA730929, PRJNA518153, PRJNA826816, PRJNA831622, PRJNA566293, and PRJEB33108; and National Microbiology Data Center at https://nmdc.cn/resource/attachment/detail/NMDCX0000148 (accessed on 10 January 2024).

## Supplementary Materials

The following supporting information can be downloaded at https://www.mdpi.com/article/10.3390/microorganisms12051030/s1: Figure S1: The ROC curve of the random forest model predicting vaginal infection; Figure S2: The ROC curve of the random forest model predicting bacterial vaginosis; Table S1: The raw data for constructing the random forest model predicting vaginal infection; Table S2: The raw data for constructing the random forest model predicting bacterial vaginosis.
