# Supplementary figures and images for: Characteristics of Vaginal Microbiota of Women of Reproductive Age with Infections
Source: Microorganisms. 2024 May 20;12(5):1030. doi: 10.3390/microorganisms12051030 (PMC11124179; doi:10.3390/microorganisms12051030)

ROC curve

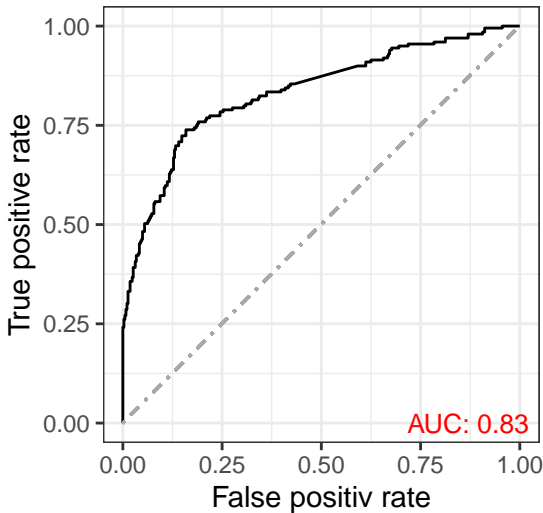

Supplement: Supplementary file 1 [file microorganisms-12-01030-s001.zip › Figure S1.pdf]

ROC curve

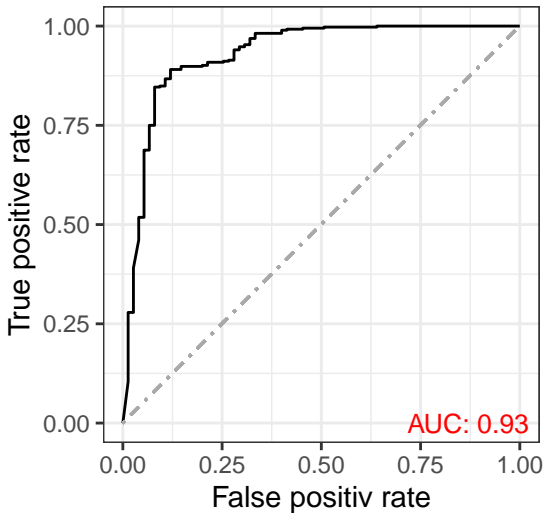

Supplement: Supplementary file 1 [file microorganisms-12-01030-s001.zip › Figure S2.pdf]
